# Relationship between land surface temperature and fraction of anthropized area in the Atlantic forest region, Brazil

**DOI:** 10.1371/journal.pone.0225443

**Published:** 2019-12-05

**Authors:** Raianny L. N. Wanderley, Leonardo M. Domingues, Carlos A. Joly, Humberto R. da Rocha

**Affiliations:** 1 Universidade de São Paulo, Instituto de Energia e Ambiente, Programa de Pós-Graduação em Ciência Ambiental, São Paulo, Brazil; 2 Universidade de São Paulo, Instituto de Astronomia, Geofísica e Ciências Atmosféricas, Departamento de Ciências Atmosféricas, Laboratório de Clima e Biosfera, São Paulo, Brazil; 3 Universidade de Campinas, Instituto de Biologia, Departamento de Biologia Vegetal, Campinas, São Paulo, Brazil; Universidade Federal de Mato Grosso do Sul, BRAZIL

## Abstract

There is growing evidence that modification of tropical forests to pasture or other anthropic uses (anthropization) leads to land surface warming at local and regional scales; however, the degree of this effect is unknown given the dependence on physiographic and atmospheric conditions. We investigated the dependence of satellite land surface temperature (LST) on the fraction of anthropized area index, defined as the fraction of non-forested percentual area within 120m square boxes, sampled over a large tropical forest dominated ecosystem spatial domain in the Atlantic Forest biome, southeastern Brazil. The LST estimated at a 30 m resolution, showed a significant dependence on elevation and topographic aspect, which controlled the average thermal regime by 2~4°C and 1~2°C, respectively. The correction of LST by these topographic factors allowed to detect a dependence of LST on the fraction of non-forested area. Accordingly, the relationship between LST and the fraction of non-forested area showed a positive linear relationship (R^2^ = 0.63), whereby each 25% increase of non-forest area resulted in increased 1°C. As such, increase of the maximum temperature (~4°C) would occur in the case of 100% increase of non-forested area. We conclude that our study area, composed to Atlantic forest, appears to show regulatory characteristics of temperature attenuation as a local climatic ecosystem service, which may have mitigation effects on the accelerated global warming.

## Introduction

Deforestation in tropical regions can be associated with ecosystem services commitment at distinct spatial scales, from carbon storage and quality of water resources to compromising regulation of surface air temperature and rainfall [1]. The Atlantic Forest is one of the most threatened biomes in South America, considered a biodiversity hotspot and a UNESCO Biosphere Reserve [2].

Understanding the roles of forests in local and regional microclimates conditions is important; however, such research is incipient and limited because of a lack of observational measurements. Land surface warming due to the conversion of tropical forests areas to another human use is mainly manifested as changes in the energy balance at a local scale due to evapotranspiration [3,4]. Indirect temperature warming occurs due to biomass carbon loss, which leads to increases in global atmospheric greenhouse gas concentrations [5]. For instance, Bala et al. [6] suggested that an increase in tropical forests cover in the 21^st^ century could contribute significantly to the reduction of global warming. Moreover, deforestation of the Amazonia can change the rainfall regime at a local scale [7] and at a regional scale, at which precipitation is likely to be reduced, disturbing climate equilibrium conditions with vegetation [8, 9].

In the processes of heat exchange from forest environments to the atmosphere and vice versa, different effects in the temperature have been noted (within versus above the canopy and during the day versus at night) compared with anthropized areas. In the Brazilian Amazonia, Culf et al. [3] measured the air temperature above the canopy and estimated an annual average warming and cooling over pasture land of approximately 2°C of the maximum and minimum daily temperature, respectively, in relation to forest land. Ewers & Banks-Leite [10] measured the air temperature at a height of 1 m outside and inside the canopies of forest fragments of the Atlantic Forest in southeastern Brazil. They showed that inside the canopy, the maximum and minimum (daily) temperatures were 40% and 60% cooler, respectively than the maximum and minimum temperatures outside the canopy. Wang et al. [11] evaluated the response of surface temperature to afforestation in a desert in Inner Mongolia. Their results showed that afforestation activities in drylands has a cooling effect of 0.5 K in the daytime in all seasons to 2006.

One method of verifying the results of energy balance processes in cases of land use changes is to assess the patterns of land surface temperature (LST), which is an estimate of the surface radiometric temperature based on the emission of thermal infrared flux captured instantaneously by a sensor at a certain measuring angle [12]. The quantification of LST temporal and spatial variability benefits from the large availability of information different spatio-temporal scales acquired by satellite platforms. For example, Landsat 8 satellite carries the Operational Land Imager (OLI) sensor with 9 spectral bands at 30 m resolution and the Thermal Infrared Sensor (TIRS) sensor with 2 thermal bands at 100 m resolution, and has a 16-day revisit frequency, whereas the Terra and the Aqua satellites carrying the MODIS sensor have a daily revisit frequency and acquire data at a coarse spatial resolution of 1 km.

Estimates of satellite derived LST have been used in multiple studies. Peng et al. [13] evaluated forest cooling in China using MODIS LST for comparison between forest to grassland and cropland. Li et al. [14] assessed biophysical effects of forests on temperature at global scale using MODIS LST to compare forest to open land, and reported daytime cooling of -4.4°C for tropical forest and -3.0°C for southern hemisphere mid latitude forest (see Tab 1 of supplementary material in [14]), which included sampling in various regions of South America. In the southern Amazonia, Nascimento [15] used the Surface Energy Balance Algorithm for Land (SEBAL) model [16,17] and LST estimates from the MODIS sensor and showed an average temperature warming of 2.5°C in pasture land compared to forest land in the dry season. Previous studies have also indicated that an increased proportion of green spaces in urban areas expressively control the LST cooling effect [18–20]. For instance, Kong et al. [20] reported that a 10% increase in tree cover area resulted in a cooling of about 0.8°C in LST in an urban region.

In addition to the effect of land cover, relief has also been reported as an important factor in temperature variation. Martin et al. [21] evaluated the variability of air temperature in regions of complex terrain in southeast Brazil and showed a diurnal air ground temperature lapse-rate of about -7°C/km. Specifically for LST, He et al. [22] investigated two small basins in mountainous areas of China that showed lapse rate ranging from -7.6 to -13.8°C/km.

The present study aims to analyze the relationship between the degree of anthropization and local temperature warming. However, to our knowledge, the relationship of LST to the fraction of anthropized area in tropical forest regions has not yet been studied. We hypothesize that areas with a lower fraction of forest cover will have higher temperatures than areas with a higher fraction of forest cover following a positive relationship between LST and the fraction of anthropized area. The analysis was carried out in an area within the Atlantic Forest region in southeastern Brazil. To test our hypothesis, we will use LST estimates derived from Landsat 5 data, on which we will apply a correction on topographic effects and analyze the relationship between the corrected LST data and the fraction of non-forested area.

## Materials and methods

### Study area

**Fig 1:**
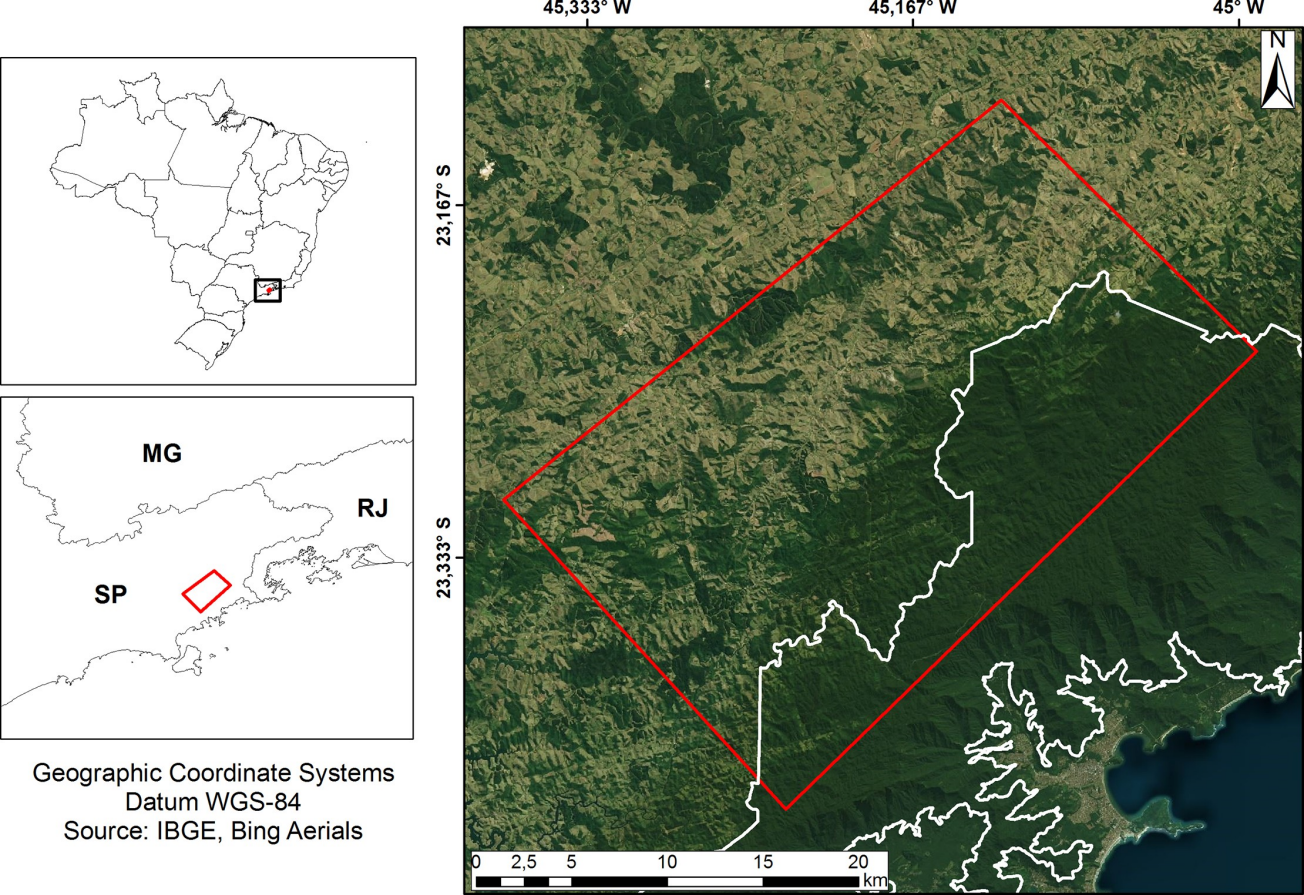
Location of the study area near the northern coast of the state of São Paulo (SP) (background image [25]). The Serra do Mar State Park is outlined in white.

A study area of 712 km^2^ was selected in the Atlantic Forest region near the northern coastal area within the state of São Paulo, Brazil, between coordinates -45.3–23.3 and -44.9–23.2 dd. The eastern sector overlaps with the Serra do Mar State Park, dominated by dense ombrophilous forest, and the northwest to northeast sector contains anthropized areas, usually small properties with pasture cover (Fig 1). The region experiences a Cfb climate (humid temperate), an annual average rainfall of 2200 mm, a humid season from October to March, and an annual average temperature of 16.5°C (Mean temperature only winter (June to September): 10°C, mean temperature only summer: 22°C (December to February)) [23,24]. This area is an anthropized region, and the forest is cleared to install pasture lands, predominance of livestock systems and it is located in a macroregion called the São Paulo Macrometropolis, one of the most populous global conurbations of about 53,000 km2 that present over 32 million hab in 174 municipalities.

### Datasets

To estimate the average LST, images from the TM sensor/Landsat-5 satellite from 2010, bands 1 (0.45 to 0.52 μm), 2 (0.52 to 0.60 μm), 3 (0.63 to 0.69 μm), 4 (0.76 to 0.90 μm), 5 (1.55 to 1.75 μm), and 7 (2.08 to 2.35 μm) all with 30 m res, and 7 (2.08 to 2.35 μm) with 120 m res, path 218 and row 76, measured at approximately 10:00 local time, with a revisit of 16 days, were used. We used eight which showed clear sky conditions over the study area. The Table 1 summarizes the Landsat images information that were retained for the LST calculation and the percentage of cloud coverage shown corresponds to the entire scene and not over the study region only.

**Table 1:** Landsat images characteristics summary used for LST calculation.

Date acquisition	% scene cloud cover	Sun elevation	Sun azimuth
2010/02/06	01	55.08	84.13
2010/02/22	04	53.19	75.42
2010/04/27	37	41.42	43.51
2010/05/13	03	38.11	39.47
2010/06/14	11	33.70	36.76
2010/08/01	04	36.92	42.24
2010/09/02	01	45.43	49.86
2010/10/20	09	59.39	70.38

The altitude and aspect maps were obtained from images of the Shuttle Radar Topography Mission 1 Arc-Second Global of the NASA EOSDIS Land Processes Distributed Active Archive Center [26] using ArcGIS software by Spatial Analyst Tools (surface > aspect). Land cover classification was determined based on a Google Earth image. The image used was dated as of year 2010 in Google Earth software and corresponds to composition of images of the year 2010. Images of this database are from Landsat and have 30m of spatial resolution (multispectral) which is pansharpened with the 15m (panchromatic) Landsat imagery.

### Methods

#### Calculation of the annual average LST

The LST was calculated following the algorithm implemented at the SEBAL model [16,17] based on the estimate of key surface variables, namely radiance, surface reflectance, emissivity, leaf area index (LAI), the normalized difference vegetation index (NDVI) and the soil adjusted vegetation index (SAVI) (shown in Tables A, B and C in S1 Appendix, [27–31]) as following:

$$LST=\frac{K_{2}}{ln(\frac{E_{NB}K_{1}}{L_{\lambda ,6}}+1)}$$

where K_1_ = 606.76 Wm^-2^ sr^-1^μm^-1^ and K_2_ = 1260.56 Wm^-2^ sr^-1^μm^-1^; $E_{NB}$ is the emissivity; *L*_*λ*,6_ is the band 6 radiance.

#### The fraction of anthropized area index (FAAI)

**Fig 2:**
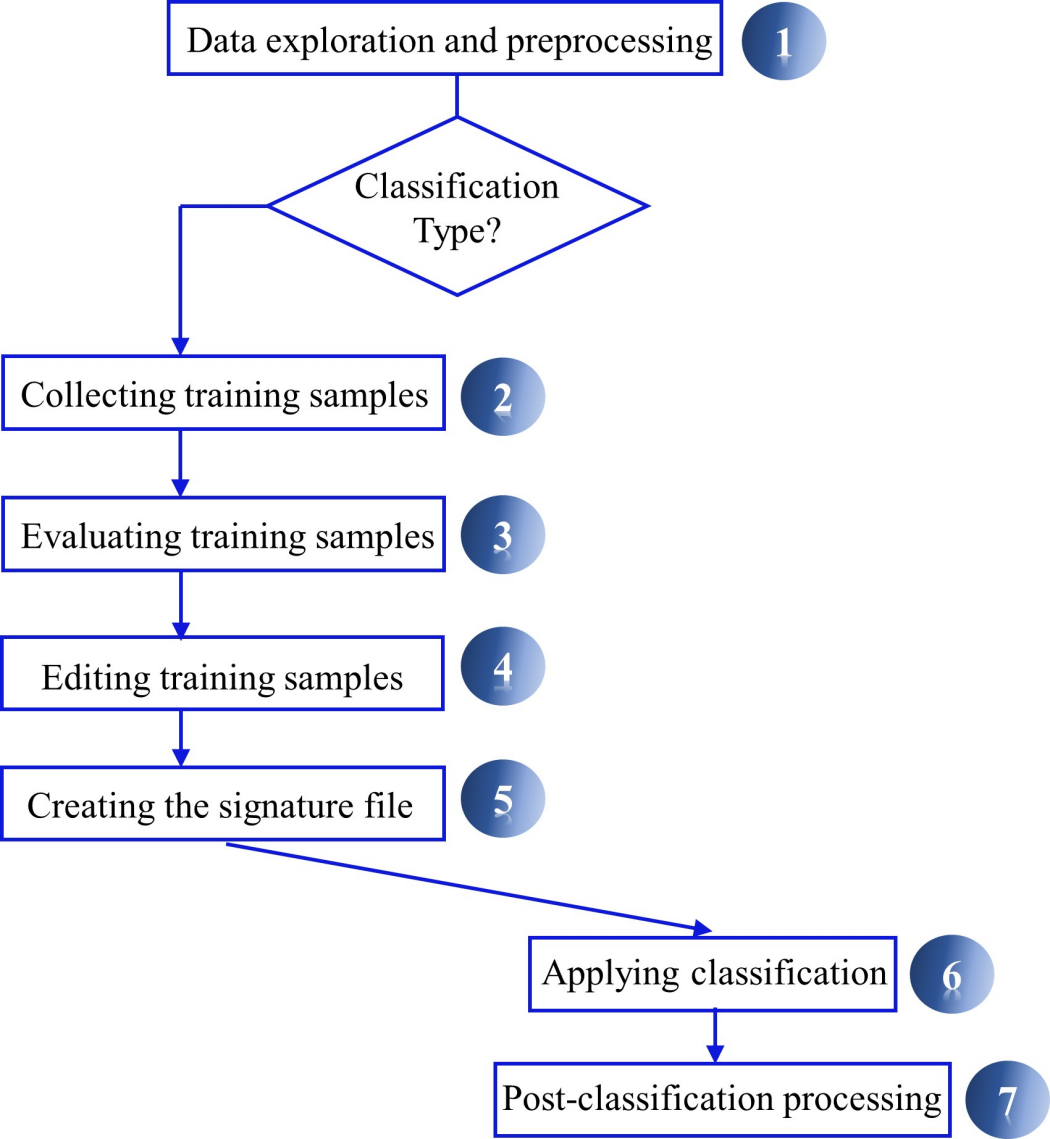
Image classification workflow (Source: Adapted from Esri).

We classified the land cover by using a supervised classification method proposed by ARSET [32] and adapted in ArcGIS version 10 (Environmental Systems Research Institute Esri, Redlands, CA, USA) (Fig 2) that uses Google Earth image with a spatial resolution of 15 m, a composite of several images from the year 2010 of the Landsat satellites and the Copernicus services. The fraction of anthropized area (FAAI) was defined simply as a non-forested area fraction, which corresponds mostly to managed pasture in the study region, and also includes very minor occurrence of small buildings, local roads and water bodies (the river Ribeirão do Chapéu and small tributary creeks). The forested areas correspond mostly to preserved native forest of the Atlantic forest biome, with very minor occurrence of eucalyptus plantation.

**Fig 3:**
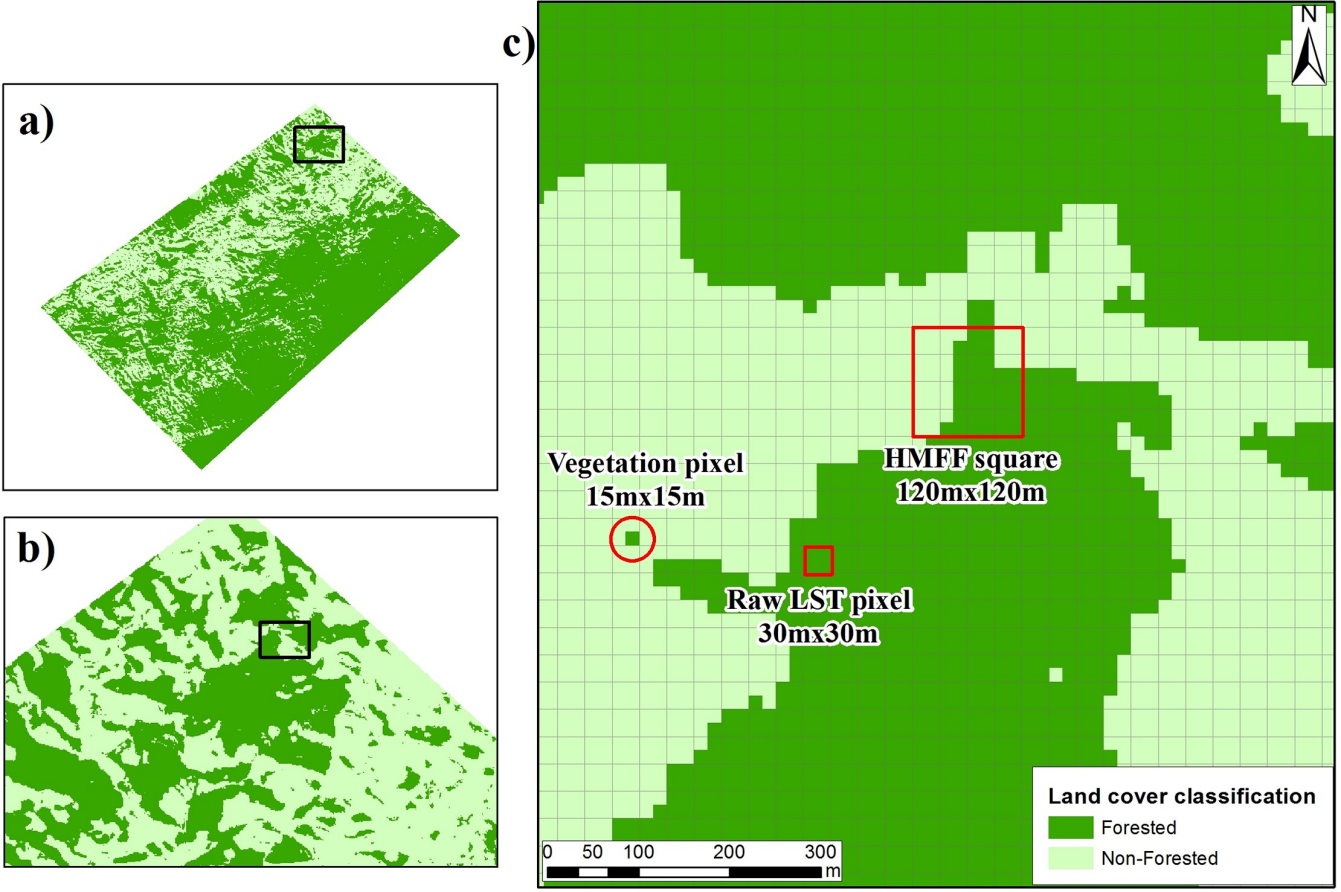
(A) Classification of land cover and the human-modified forest fractional area (FAAI) index, where the black squares in (a) and (b) are magnified in (b) and (c), respectively. The degradation of spatial resolution is shown in (c), where square of 15 m × 15 m defined land cover (red circle), squares of 120 m × 120 m were used to calculate FAAI (large red square), and squares of 30 m × 30 m were used to calculate LST (small red square).

We accounted 78,455 samples of forest and 18,792 samples of non-forested at 15 m resolution using photointerpretation, where the forest samples showed dark green color and pasture light green or light red, respectively, with an interactive supervised classification tool. We applied a pos-classification process to reduce the salt-and-pepper effect in the output classified image, using the majority filter and maximum likelihood classification tools [33,34]. Ultimately the land cover was classified as forested and non-forested (Fig 3A), with an accuracy of 98.8%.

The squared window used to upscale/resample the land cover data at 15 m resolution was empirically fixed at a 120 m length. The fraction index of anthropized forest area was defined as the fraction of anthropized area index (FAAI), which was equal to the percentage of the occurrence of non-forested area (specified in 15 m × 15 m squares) within an area of 120 m × 120 m (Fig 3). In other words, each square contained 64 squares with a 15 m × 15 m resolution with prescribed land cover (14,400 m^2^), where, for example, the occurrence of 16 non-forest squares represented an FAAI of 25%. We decided for a minimum scale that could reduce potential sources of error (e.g. shadowing effect of tropical trees in resolution grids with 30 m and 60 m) and concurrently provide a maximum scale sufficient to describe a large number of classes for FAAI variation. For example, with the 90 m scale the variation of FAAI is about 2.7%, while for 120 m it is about 1.5%, which is sufficiently large to explain the variability we propose to investigate. Also, the computational cost of data processing was substantially reduced at 120 m scale when compared to 90 m, by about 44%.

#### Relationship between LST & FAAI before and after topography effect correction

The annual average LST was calculated from the 8 selected images at a 30 m resolution. The 30 m resolution LST is then spatially upscaled by calculating the average LST in the 14,400 m^2^ squares, totaling 49,457 squares. LST was later corrected for terrain topography (altitude and aspect, respectively) to relativize these effects and emphasize the control of land cover on the spatial variability of LST. Evidence of field observations over a complex terrain near our region of study and with similar relief´s characteristics [22] showed how ground air temperature decrease with altitude and land surface temperature shows maxima at mountain places with aspects facing north.

**Fig 4:**
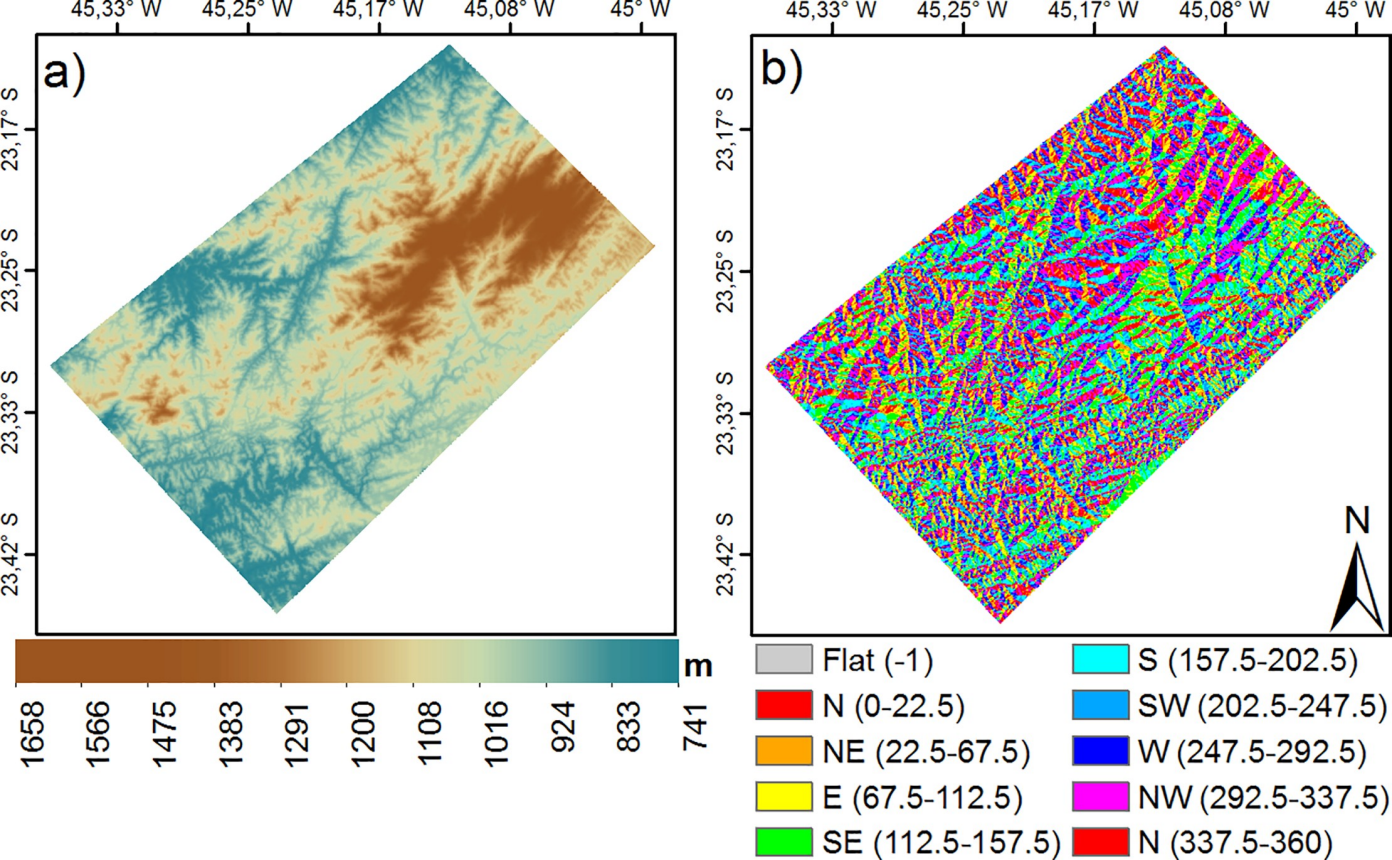
(a) Altitude (m), (b) slope aspect (°).

The altitude in the study area (Fig 4A) varies from approximately 740 to 1650 m, with complex terrain featured in the east with heights above 1000 m. The aspect of the region (Fig 4B) exhibits the ridges of hills (the line dividing slopes of approximately opposite aspects) aligned with the coastline (approximately southwest–northeast) as an organized pattern. As such, slopes tend to face southeast or northwest.

We estimated the effects of altitude, *h*, and aspect, *a*, to calculate corrected surface temperatures as T_c_ = T_R_−(ΔT_*h*_ + ΔT_*a*_*)*, where T_R_ is the raw surface temperature, ΔT_*h*_ and ΔT_*a*_ are the differences of T_R_ to the reference surface temperature averaged each FAAI interval, with the reference for altitude taken at 850m and for aspect at 45^o^, respectively. The differences were estimated with statistical polynomial fitting that used data at discrete intervals of every 50 m for the altitude, 20° for the aspect and 10% for the FAAI variation, respectively. Corrections were applied to all data at 120 m res, that provided information at each square grid element for different combinations of altitude and FAAI, and aspect and FAAI. A detailed description is given in S2 Appendix.

We used McNemar’s statistical test to estimate significance of the differences in performances of the two temperature fitted models (raw and corrected), which uses the Chi-squared test for goodness of fit. It is applied to a 2×2 contingency table, the cells of which include the number of paired samples (raw and corrected) with absolute residuals lower than their standard error of regression (approved), or higher (disapproved), namely: a_11_ = both approved; a_12_ = only raw approved; a_21_ = only corrected approved; a_22_ = both disapproved.

## Results and discussion

**Fig 5:**
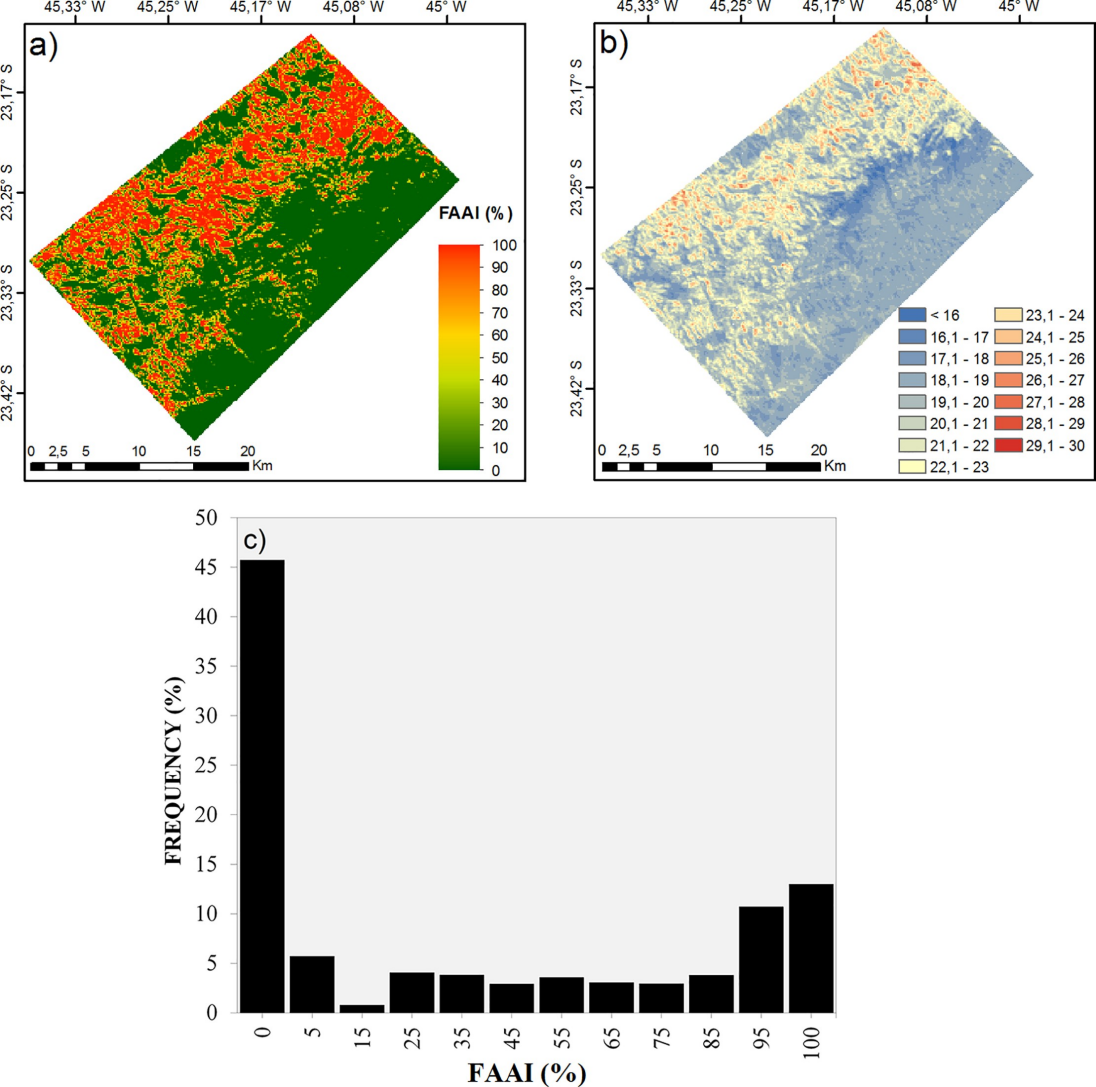
(a) Average FAAI at a resolution of 120 m (%). (b) Average LST at a resolution of 120 m (°C). (c) Histogram of FAAI occurrence by intervals of 0% to 5%, and then from 5% to 95% in 10% increments, and finally from 95% to 100%.

The mean calculated FAAI at 120 m res (Fig 5A) indicated the dominance of FAAI values below 10% with the presence of forests in the south and east sectors corresponding to the Serra do Mar State Park, as anticipated from the classification presented in Fig 3, and non-forested squares (FAAI > 0%) in the west and north sectors. The average LST (Fig 5B) generally varied from 15 to 28°C, indicative of colder temperatures in areas with low FAAI values (0–10%). The areas presenting less than 10% of FAAI were 1.5°C colder than the areas with more than 90% of FAAI. The FAAI distribution showed that approximately 46% of squares were classified as completely forested (FAAI = 0%) and 13% were classified as completely anthropized (FAAI = 100%) (Fig 5C). Cases of partial anthropization accounted for 41% and were reasonably well distributed along the intervals (Fig 5A). Cases of partial FAAI (> 10% and < 90%) did not occur in isolated blocks, but as transition spaces between the FAAI blocks of 0% or 100% (Fig 5A).

**Fig 6:**
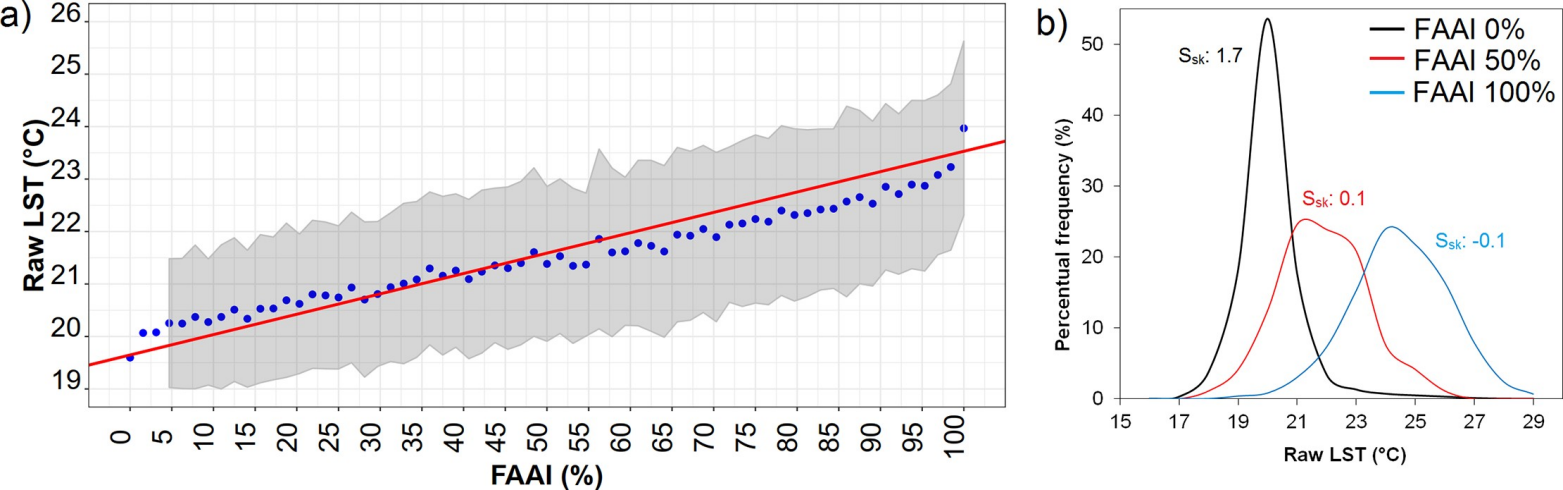
(a) Relationship of raw LST (°C) with FAAI (%) (blue circles are average LST at each FAAI class; grey shaded area is bounded by standard deviation of LST per FAAI class; red line is linear regression of all LST data against FAAI, LST = 0.03 FAAI + 19.6; R^2^ = 0.6, significant p-value < 2.2e^-16^); and (b) histogram of the LST distribution at each FAAI class 0%, 50% and 100% and associated skewness coefficient (S_sk_).

The raw (uncorrected) LST at 120 m res showed a direct positive association with FAAI (S_sk_: 0.8), with an asymmetrical distribution (mean: 20.9°C, median: 20.2°C, mode: 19.5°C, sd: 2.0°C). LST variability was higher for the squares containing only one class (i.e. 0% FAAI and 100% FAAI, corresponding to 100% and 0% non-forested squares, respectively) since they are widespread over the study region (Fig 5A and 5C). To test the effect of the non-forested area fraction on LST locally we upscaled the 30m res information to 120 m res in order to get more variability along the FAAI range. The pattern of increasing LST with FAAI initially confirmed our hypothesis (Fig 6A); however, the quantification of raw LST with FAAI variation could be improved due to the need for correction of the dependence of temperature on topography.

### Topography effects correction

**Fig 7:**
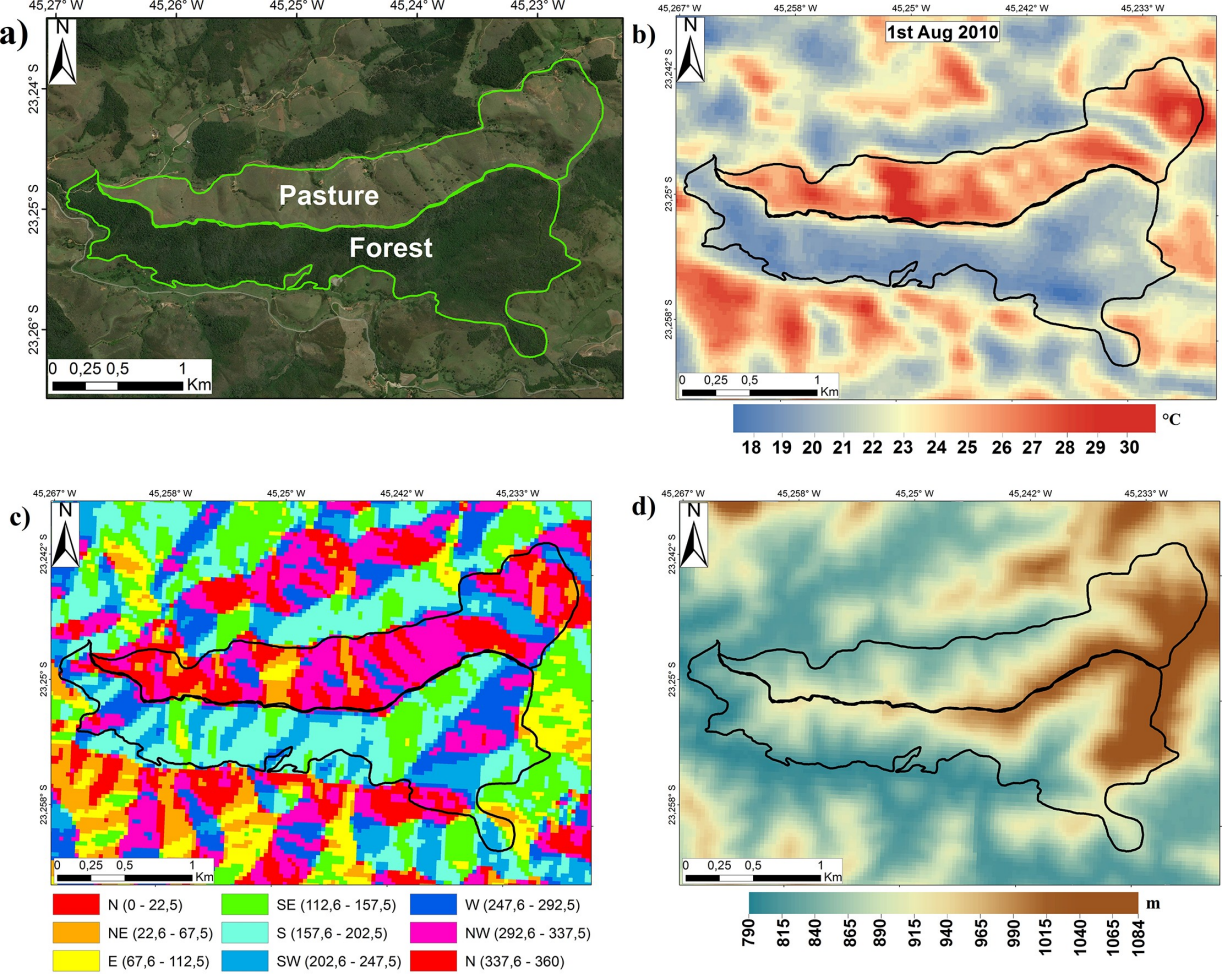
(a) Forest vegetation patch to the south of a pasture area (perimeters outlined in light green) (source: Microsoft Bing Maps Services) near Catuçaba district, São Luiz do Paraitinga, SP, with associated information of (b) LST (°C) (August 1, 2010), (c) aspect (°), and (d) altitude (m).

A suitable example of the dependence of temperature with topography in our study domain is illustrated over an area with forest vegetation fragment in contact with grass (pasture) (Fig 7A). The pattern of the calculated LST showed differences of daytime temperature of about 10°C in August, with cold temperatures (≃20°C) in the forest and warm temperatures (≃30°C) in the pasture (Fig 7B). However, the aspect spatial distribution showed more southern slopes in the forested area, and slopes facing northward in the pasture (Fig 7C), which partially explained the temperature difference, since north-facing slopes receive more insolation than south-facing slopes at this time of year. Such differences were also noted at smaller scales of the domain e.g. the small strips of south-facing slopes in the pasture area were colder than the north-facing surroundings.

Although air temperature generally varies with altitude, this example suggests how the isolation of altitudinal effect needs to be properly investigated, both because the area had a relatively modest altitude variation (~250 m; Fig 7D) and because it was located in a matrix with distinct aspect and cover vegetation opposite.

Thus, this case exemplifies the possible aggravation of LST estimation in pasture areas due to differences in incoming radiation. However, throughout the whole study area, various potential cases could be expected, such as the case in which the LST in anthropized areas was reduced by negative compensations due to aspect. Such complex interactions require that all aspect and anthropized areas be analyzed together.

**Fig 8:**
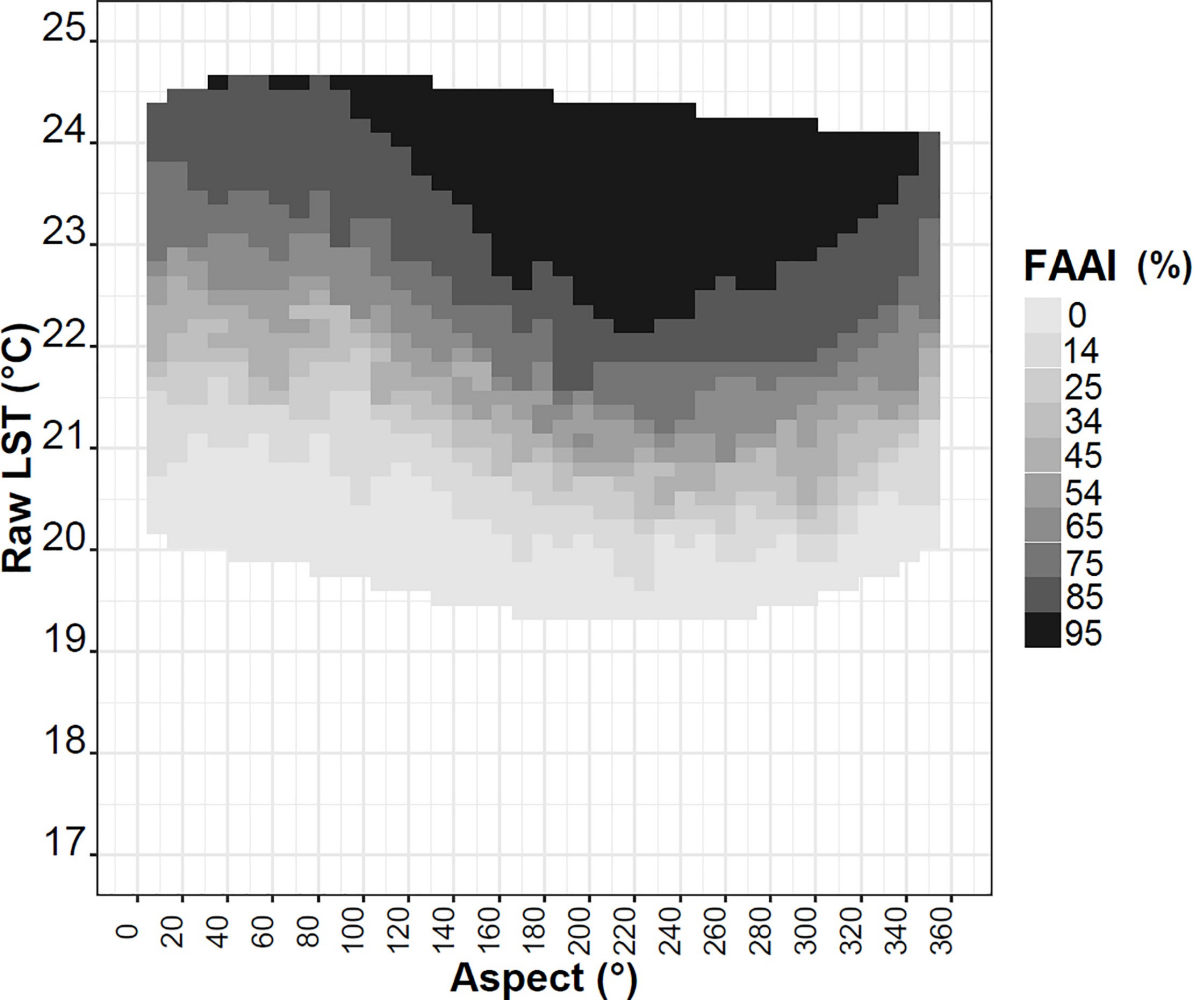
Average raw LST (°C) depending on the terrain aspect (in classes of 5°) and for FAAI classes (5–95%). The gray scale is the median of each FAAI class (%).

To effectively evaluate the influence of terrain aspect on temperature, the average raw LST scattering was shown by distinct classes of aspect and anthropization (Fig 8).

In this visualization, cold and hot temperature bands clearly corresponded respectively to smaller and larger FAAI values, as expected, although they varied differently according to specific FAAI class and aspect. For instance, the temperature range due to the variation in aspect was approximately 2°C in the highest FAAI classes but around 1°C in the lower classes. In particular, in the 95% FAAI class, the amplitude due to aspect was 2.7°C (Table 2), while at 5%, it was 1.1°C (Table 2). These differences in amplitude suggest that the calculated LST was more sensitive to aspect in areas with less forest cover (FAAI > 90%), where temperature warming was also higher. The directional variability in lapse rate was also showed by He et al. [23], associating the higher lapse rate at locals with aspect from east to southwest, showing that this range the aspect is favored by incident solar radiation.

**Table 2:** Absolute maximum amplitude of LST (maximum LST minus minimum LST,°C) median (± SD) by FAAI class (%) for variation of aspect and altitude respectively.

	Aspect		Altitude	
FAAI (%)	amp_max_ (°C)	median ± SD	amp_max_ (°C)	median ± SD
5	1.1	19.6 ± 1.1	2.7	19.0 ± 0.8
15	1.9	20.4 ± 1.3	3.0	19.6 ± 1.0
25	1.6	20.7 ± 1.3	3.1	19.9 ± 1.1
35	2.1	21.2 ± 1.4	2.9	20.2 ± 1.2
45	2.1	21.3 ± 1.4	3.4	20.4 ± 1.3
55	2.3	21.5 ± 1.4	3.4	20.6 ± 1.3
65	2.1	21.9 ± 1.5	3.7	20.9 ± 1.4
75	2.1	22.2 ± 1.4	3.4	21.3 ± 1.5
85	2.2	22.5 ± 1.4	3.8	21.7 ± 1.4
95	2.7	23.5 ±1.4	2.8	22.9 ± 1.6

The general dependence of LST on aspect showed a pattern of maxima in north-facing aspects (aspect 292.5 to 67.5°). This preferred direction favoring temperature warming could be explained physically by the greater amount of solar irradiance during most of the year, but in particular during March–December when the solar angle is positioned to the north. Where the LST minima were established, the aspect classes were also well defined and coherent with the solar angle, as they were generally located approximately in southwest-facing slopes (aspects of 220° and 260°) for all FAAI classes (Fig 8). Moreover, these LST minima exhibited particular variations according to FAAI class. For high FAAI classes, the aspect associated with the minimum LST was somewhat lower compared with low FAAI classes.

**Fig 9:**
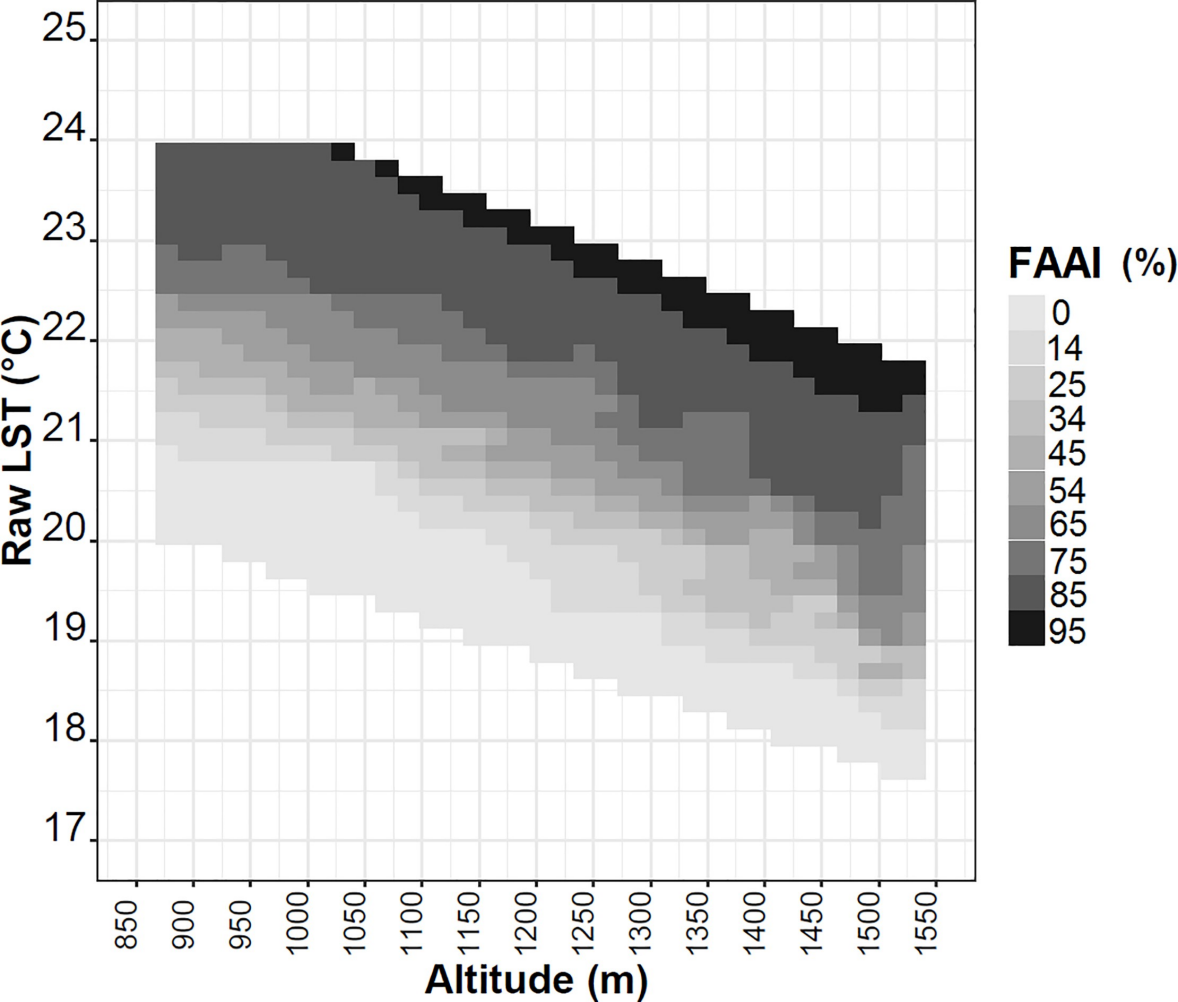
Average raw LST (°C) by altitude (m) among FAAI classes. The gray shading represents the median of the FAAI class (%).

A significant systematic cooling pattern of the average raw LST was observed with altitude for all FAAI classes (Fig 9). In this figure, cold and hot LST corresponded to low and high FAAIs, respectively, which suggests that areas with higher forest cover percent show lower temperature in accordance with the previously results obtained [13,14,32]. The relationship of LST with altitude was approximately linear, except at extremes of altitude above 1500 m, where a saturation is noticed (Fig 9).

The temperature range with regard to the variation in altitude was between 2.7°C and 3.8°C along approximately 700 m of altitudinal change (Table 2), which was higher than the amplitude with regard to aspect (Fig 7), and no noticeable control of anthropization was observed. The statistically fitted equations of the raw LST with the topographic variables are shown in S2 Appendix, which indicate proportions of LST variation with altitude range in all analyzed FAAI classes, the lapse rate ranged from -3.9 to -5.2°C/km, leading to a cooling of the LST with altitude at 10:00 local time. This result is in accordance with Minder et al. [35], and also showed lower rates than the daytime terrestrial lapse-rate measured with thermometers at 2 m above ground level in a nearby mountain region by Martin et al. [21].

### Relationship between LST and the fraction of anthropized area after topography effects correction

**Fig 10:**
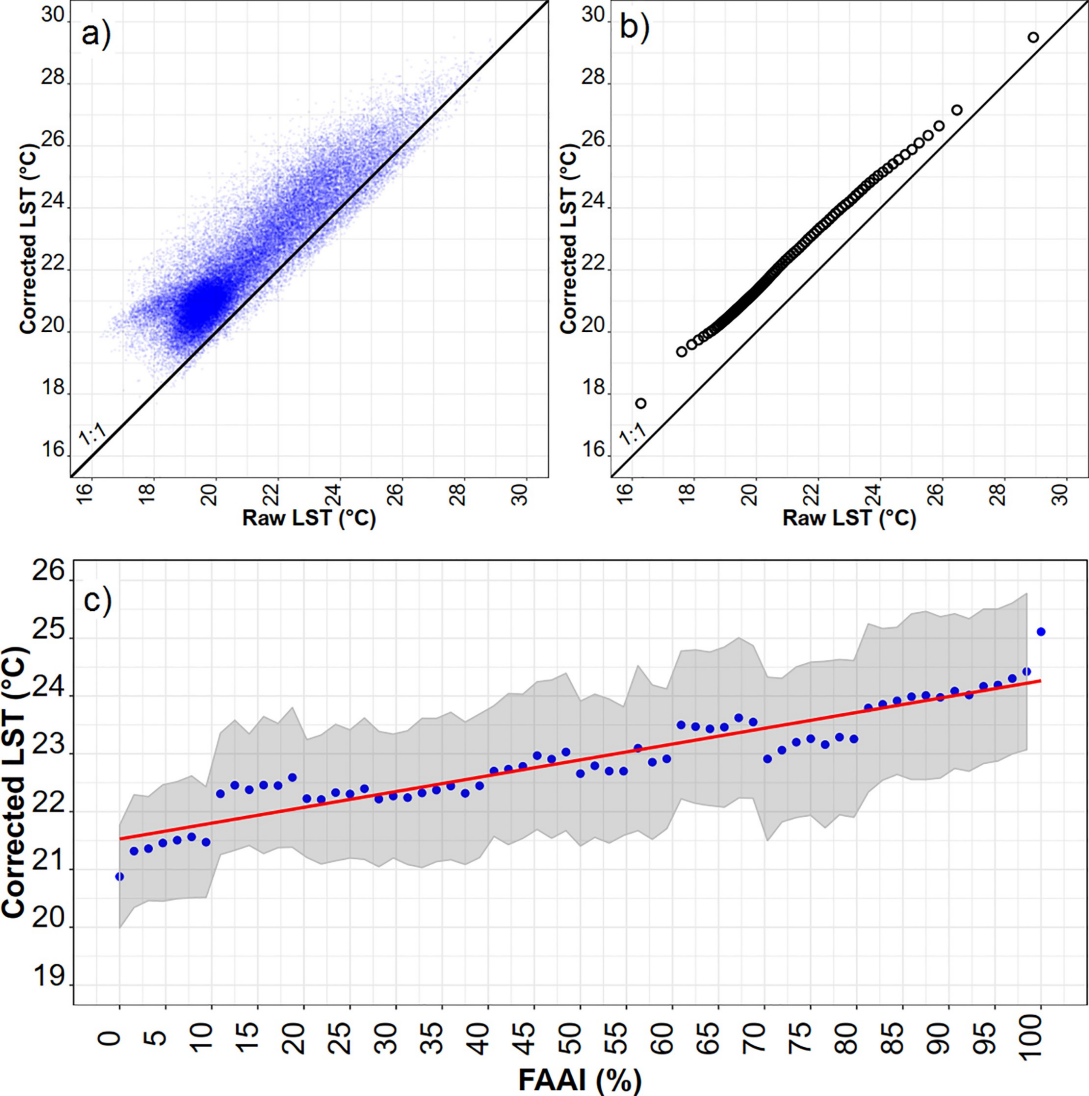
(a) Scatterplot of raw LST and corrected LST (y = 4.7 + 0.83·x); R^2^ = 0.82, significant p-value < 2.2e^-16^). (b) Quantile–quantile distribution of raw LST and corrected LST. (c) relationship of corrected LST with FAAI (%) (blue circles are average LST at each FAAI class; grey shaded area is bounded by standard deviation of LST per FAAI class; red line is linear regression of all LST data against FAAI, LST = 0.038 FAAI + 20.9; R^2^ = 0.63, significant p-value < 2.2e^-16^).

The corrected average LST removed the apparent effects of topographic aspect and altitude from the raw LST. The extremes of corrected temperature were respectively equal to 17.8°C and 29.8°C, or an amplitude of 12°C (Fig 10A and 10B). The raw data generally underestimated the corrected information (Fig 10A), where the correction led to an average increase of 1.4°C. The statistical distribution in quantiles showed that the greatest discrepancy occurred in colder temperature ranges (< 19°) and that the deviation tended to decrease at warmer temperatures (Fig 9B). The final correction resulted in an increase in temperature, which seemed reasonable given the relationships of raw LST with altitude and aspect, and since we assumed a lower altitude than the original as a reference for the correction (conditioned to the FAAI class variability). In addition, aspect correction generally increased temperature because the aspect of reference was roughly northeast (45°), while the other points were southward (112° to 247.5°). The greater spread around cold temperatures was partly associated with the sampling areas at higher altitudes, near mountain ridges, where the uncertainty of the aspect determination was higher; therefore, the correction resulted in greater variance.

Previous investigations of forest LST cooling used less accurate spatial resolution and corrections for the topography effects. Li et al. (Supplementary Fig 4 in [14]) corrected for elevation adjustment with sampling constraint to differences within ±500 m that totaled 11,530 valid cells at 5 km res. Peng et al. [13] reduced sampling to elevation differences in the range ±100 m, that resulted in 671 paired valid grid cells at 1 km res (Supplementary Figure 20 in [13]). Our method improved the approach while using 30 m spatial resolution for both corrections of elevation and aspect, taken on the whole altitudinal range, that resulted in the greater amount of 49,457 valid cells to discuss the LST cooling effect.

In order to analyze whether the differences in the fitted model accuracies produced by the corrected and the raw LST, respectively, were statistically significant, we applied a two-tailed McNemar´s test [presented in Table D in S1 Appendix], which confirmed that the corrected model significantly improved in comparison to the raw model (χ2 = 9.6, p-value < 0.001 at 95% confidence interval).

The corrected LST revealed a significant linear increase in temperature with the degree of forest anthropization in a rate of +3.8°C per 100% of FAAI variation (Fig 10C). At the extremes of FAAI we noted a large variance of the corrected LST.

The fitted cooling of -3.8°C associated to FAAI = 0% appears to be consistent to similar findings of mean cooling as shown by Li et al. [14] of -4.4°C for tropical forest compared to open land (daytime sampling averaged between 20°S to 20°N), and of -3.0°C for southern hemisphere mid latitude forest to open land (id. 20°S to 50°S) (Supplementary Table 1 in [14]). Our estimate of cooling rate is approximately intermediate between those author´s estimates of tropical and mid latitudes. Despite our analysis is not strictly comparable to those authors, our site is located in the transition of tropical to subtropical zones, what might help to explain keeping the consistency of the two studies.

The attribution of LST to forest land cover variability by high-resolution satellite data represents a modern research technique that is viable under proper conditions, such as access to data with an adequate resolution and sufficient statistical sampling. However, the results may depend on a number of factors associated with surface cover, which could be effectively exploited *posteriori* to yield more accurate results.

For instance, the present study had several limitations: the uncertainty of the prescription of vegetation cover at a sub-pixel resolution, which, despite the 30 m resolution, could be improved; the size of the statistical grouping of squares to form a representative element of the fraction of anthropization; and the greater detailing of land cover classes in the vegetation supervision stage, which may discern subclasses with particular LST response patterns (forests with different characteristics, forests in different stages of succession, pastures under different management conditions and levels of degradation and plant productivity, crops with different management and stages of growth, and areas with buildings in the rural area).

## Conclusions

The pattern of daytime surface warming in anthropized areas of tropical forests, at local scale such as that measured meteorologically above the canopy in the Amazon [3] or evaluated by LST [13,14], was determined using estimated LST from satellite at 120 m resolution upscale. The analysis was carried out in an area within the Atlantic Forest region in southeastern Brazil, located in a macroregion experiencing high strategic socioeconomic and population growth, making it vulnerable as dependent on ecosystem services. There is a sense that LST increases when non-forest lands are compared to forests, based on the biophysical cooling forest mechanism. It is often shown comparisons of areas fully covered with forests to areas fully covered with a specific altered ecosystem (e.g. crop, grass, shrubland, urban). There is vast literature confirming such sense by using numerical modelling, often methods of climate simulations with surface-vegetation transfer schemes coupled to atmospheric models, e.g. the cases of tropical deforestation in Amazonia [8,9]. Secondly, there is scarce literature reporting this sense using actual field observations, what is not surprising, as it requires expensive and complex methods that measure accurate above canopy hourly basis air temperature at the top of tall micrometeorological towers placed at paired sites under a common large scale climate. We noted [3] as the only proper example of field study in Amazonia and, to our knowledge, none exists in other tropical forests. Finally, growing literature has used information of satellite surface temperature, that basically shows an averaged forest cooling effect using deviations at a pixel-to-pixel basis comparison over sampling that has either full forest or full altered ecosystem cover, e.g. [11,13,14,15] as recent meaningful studies. We noted [20] meant an improvement in using satellite temperature for these studies, who suggested that a 10% increase in tree cover area resulted in an 0.8°C cooling in a densely urbanized region, a result however potentially biased with the use of only one satellite image and lack of topography effect correction.

We attempted to innovate the existing knowledge by moving beyond and into the local scale variability with simple methods. The question is if land use variability of increasing anthropized vegetation within a small spatial domain can be detected in order to show consistent warming rate proportional to the fraction of anthropized area when integrated over a large domain. The method used remote sensing data and searched for statistically significant results whereupon improving the accuracy relatively to previous studies.

The spatial variability of LST was partly explained by both the altitude and aspect under the conditions of this study. The LST variations were generally due to aspect in the range of 1 to 2°C, while due to altitude (within the 700 m variation covered in this study) were between 2 to 4°C. The dependence of LST to the topography could be caused in part simply by different forested land cover at ridges compared to valleys and hillslopes, but it was shown to be caused mostly by the vertical atmospheric variability and face direction oriented hillslopes.

The results support a strong dependence of LST on the degree of anthropization of forest cover, which was estimated as the fraction of non-forested area detected by pixel and LST sampling at high resolution (30 m) and quantified at upscalled lower resolution (120 m). The relationship showed an approximated linear increase in surface temperature with increasing degree of non-forested area, whereby approximately each 25% areal increase of non-forested cover resulted in 1°C of surface warming. From this average projection, the maximum temperature warming would occur as about 4°Cover a 100% non-forested area.

We recognize how other factors can potentially control the surface temperature, that could not be included properly in our estimates due to the limited information provided by our set of satellite images. Our findings were constrained by weather conditions at the middle morning time and clear sky days, similar to previous findings [13,14], a simplification that opens various other ways to help improving the estimates by using higher spatial and temporal resolution information, namely: the influence of the temporal variability of the surface energy balance caused by the seasonality of key variables as green leaf area index and soil moisture; the spatial sub-pixel variability, which includes the accountability of other land cover types likely to occur significantly at preferential areas, e.g. crops, irrigated areas, planted forests, and urban areas; the temporal variability of energy partition (between evapotranspiration and heat flux) day around; and finally how the occurrence of clear sky days prevailed in our calculation, as opposed to cloudy days, that were not taken into account. For example, it is expected that the differential daytime warming over forest and non-forest areas is less in cloudy days compared to clear days as the available energy is generally reduced.

We conclude the forest land in this region of the hot spot Atlantic Forest biome can act to attenuate local surface temperature in rural areas as a climatic ecosystem service. This, in turn, helps to explain forest fragmentation effects that are supposed to be relevant to species conservation, with losses of ecosystem services brought about deforestation still unknown. This is also expected to bring climatic control on regional remote areas that mitigate the effects of urban heat islands, and/or to adapt to the long term global warming effect, although this requires scientific validation because these effects depend on regional/synoptic atmospheric variability and other local controls, for which there is not comprehensive studies so far.

## Supporting information

S1 Appendix Table of equations of the variables used to LST calculate and Table of McNemar’s Chi-squared. (DOCX)

S2 Appendix Equations used to correct the land surface temperature. (DOCX)
