# Supplementary material for: Relationship between land surface temperature and fraction of anthropized area in the Atlantic forest region, Brazil
Source: PLoS One. 2019 Dec 5;14(12):e0225443. doi: 10.1371/journal.pone.0225443 (PMC6894832; doi:10.1371/journal.pone.0225443)
Supplement: S1 Appendix — (DOCX) [file pone.0225443.s001.docx]

**S1 APPENDIX**

The equations used to estimate the fundamental variables and the land surface temperature (LST) as used in the Surface Energy Balance Algorithm for a Land (SEBAL) are shown in Table A.

**Table A.** Fundamental variables, and equations used in the SEBAL model for the TM sensor (Landsat-5) for the LST calculation.

| **Variable** | **Equation** | **Details** |
| --- | --- | --- |
| Radiance ($\text{L}_{\text{λ}}$) | $\left( \frac{\text{L}_{\text{MAX}}\text{-}\text{L}_{\text{MIN}}}{\text{255}} \right)\text{DN+}\text{L}_{\text{MIN}}$ | L_MAX_ and L_MIN_: spectral calibration constants of the sensor (Table B); DN: digital pixel number |
| Reflectance (ρλ)^[27]^ | $\frac{\text{π}\text{L}_{\text{λ}}}{\text{ESUN}_{\text{λ}}{\cos\text{θ}\text{d}}_{\text{r}}}$ | ESUN_λ_: monochromatic solar constant (Table C); θ: solar zenith angle; d_r_: relative Earth–Sun distance. |
| Relative Earth–Sun distance (d_r_) | $\text{1+0}\text{.}\text{033cos}\left( \text{DOA}\frac{\text{2π}}{\text{365}} \right)$ | DOA: day of the year |
| SAVI ^[27,28]^ | $\frac{\left( \text{1+L} \right)\text{(}\text{ρ}_{\text{I}\text{R}}\text{ -}{\text{ }\text{ρ}}_{\text{R}}\text{)}}{\text{(L+}\text{ρ}_{\text{I}\text{R}}\text{ + }\text{ρ}_{\text{R}}\text{)}}$ | L= 0 (dense vegetation), 1 (sparse vegetation), 0.5 (intermediate vegetation) |
| NDVI ^[29,30]^ | $\frac{\text{ρ}_{\text{IV}} \text{-}{\text{ }\text{ρ}}_{\text{R}}}{\text{ρ}_{\text{I}\text{R}}\text{ + }\text{ρ}_{\text{R}}}$ | IR: infrared; R: red |
| LAI | $\frac{\ln\left( \frac{\text{0}\text{.}\text{69 - SAVI}}{\text{0}\text{.}\text{59}} \right)}{\text{0}\text{.}\text{91}}$ |  |
| Emissivity (ε*_NB_*) | 0.97 + 0.0033 LAI |  |

In order to calculate the LST, it was necessary to correct the radiances with the spectral calibration for the bands based on the constants L_min_ and L_max_ (Table B) and to correct the reflectance with the monochromatic solar constant (Table C).

Table B: Spectral calibration constants (L_MAX,_ L_MIN_) to Thematic Mapper sensor Landsat 5 [31].

| **Band (**𝛌**)** | **L_MIN_ (Wm^-2^st^-1^μm^-1^)** | **L_MAX_ (Wm^-2^st^-1^μm^-1^)** |
| --- | --- | --- |
| 1 | -1.52 | 193.0 |
| 2 | -2.84 | 365.0 |
| 3 | -1.17 | 264.0 |
| 4 | -1.51 | 221.0 |
| 5 | -0.37 | 30.2 |
| 6 | 1.2378 | 15.303 |
| 7 | -0.15 | 16.5 |

Table C: Monochromatic solar constante values (ESUNλ) (Wm-2μm-1) to Landsat 5 – TM (Chander e Markham, 2003[31]).

| **Band 1** | **Band 2** | **Band 3** | **Band 4** | **Band 5** | **Band 6** | **Band 7** |
| --- | --- | --- | --- | --- | --- | --- |
| 1957 | 1826 | 1554 | 1036 | 215.0 | - | 80.67 |

Table D: Table of McNemar’s Chi-squared applied to our sampling to estimate significance of the differences in performances of two temperature fitted models (raw and corrected).

| LST Corrected | | | | |
| --- | --- | --- | --- | --- |
| LST Raw |  | Approve | Disapprove | Σ |
|  | Approve | 31,396 | 5,166 | 36,562 |
|  | Disapprove | 5,487 | 7,408 | 12,895 |
|  | Σ | 36,883 | 12,574 | 49,457 |
