# Supplementary material for: Relationship between land surface temperature and fraction of anthropized area in the Atlantic forest region, Brazil
Source: PLoS One. 2019 Dec 5;14(12):e0225443. doi: 10.1371/journal.pone.0225443 (PMC6894832; doi:10.1371/journal.pone.0225443)
Supplement: S2 Appendix — (DOCX) [file pone.0225443.s002.docx]

**S2 APPENDIX**

We estimated equations to correct the surface temperature for the topographic effects obtained from polynomial regressions of the raw temperature with altitude *h,* ($\hat{T}_{h}$) and with the aspect *a*, ($\hat{T}_{a}$). The corrected surface temperature was T_c_ = T_R_ – (ΔT*_h_* + ΔT*_a_)*, where T_R_ is the raw surface temperature, ΔT*_h_* and ΔT*_a_* are the differences of T_R_ to the reference surface temperature averaged each FAAI interval, with the reference for altitude taken at 850 m and for aspect at 45^o^, respectively. The differences were estimated with statistical polynomial fitting that used data at discrete intervals of every 50 m for the altitude (Table 1), 20° for the aspect (List 1) and 10% for the FAAI variation, respectively. In summary the differences were calculated as ΔT_h_ =$\hat{T}_{h,FAAI}-\bar{T}_{850m,FAAI}$, where $\bar{T}_{850m,FAAI}$ is the average temperature at 850 m each FAAI interval; and ΔT_a_ =$\hat{T}_{a,FAAI}-\bar{T}_{45^{\circ},FAAI}$. where $\bar{T}_{45^{\circ},FAAI}$ is the average temperature at 45° each FAAI interval. Corrections were applied to all data at 120 m res for different combinations of *h* and FAAI, and *a* and FAAI.

**Table 1.** Equations fitted for altitudinal variation and dependence on FAAI (taken at the middle interval, in %): $\hat{T}_{h}$ (°C) = b (°C⋅100m^-1^) ∙ h (altitude, m) + a (°C).

| **FAAI (%)** | ${\hat{\boldsymbol{T}}}_{\boldsymbol{h}}$ | **R^2^** | **p-value** |
| --- | --- | --- | --- |
| 5 | -0.39 h + 23.7 | 0.97 | 5.8e^-12^ |
| 15 | -0.46 h + 25.2 | 0.97 | 6.8e^-12^ |
| 25 | -0.43 h + 25.2 | 0.96 | 2.9e^-11^ |
| 35 | -0.44 h + 25.6 | 0.98 | 3.9e^-13^ |
| 45 | -0.46 h + 26.1 | 0.96 | 8.0e^-11^ |
| 55 | -0.43 h + 25.9 | 0.94 | 2.0e^-10^ |
| 65 | -0.48 h + 26.7 | 0.91 | 7.3e^-09^ |
| 75 | -0.44 h + 26.6 | 0.89 | 3.8e^-08^ |
| 85 | -0.52 h + 27.8 | 0.93 | 3.6e^-09^ |
| 95 | -0.43 h + 28.0 | 0.95 | 1.9e^-09^ |

**List 1:** Equations fitted for variation of aspect, *a*, and dependence on FAAI (taken at the middle interval, in %) $\hat{T}_{a}$ (°C) = f (*a ^n^*) as a polynomial fitting (degree n = 4 or 5), with $\hat{T}_{a}$ = y and *a* = x as following**:**

**5%:** y = 1.61779094∙10^-12^x^5^ – 2.33860247606∙10^-9^x^4^ + 1.25591999175501∙10^-6^x^3^ - 2.66671263506879∙10^-4^x^2^ + 0.0153885560872595x + 20.0653840278255
R² = 0.98, p-value: < 2.2e^-16^

**15%:** y = -3.0794805345∙10^-10^x^4^ + 4.6282823936653∙10^-7^x^3^ – 1∙53386952271406∙10^-4^x^2^ + 9∙666318403319∙10^-3^x + 21.0685572245873

R² = 0.84, p-value: 4.2e^-12^

**25%:** y = -5∙0863959982∙10^-10^x^4^ + 6∙0917545530951∙10^-7^x^3^ – 1.97423557920073∙10^-4^x^2^ + 0.0159021036803466x + 21.0782574462278

R² = 0.86, p-value: 3.9e^-13^

**35%:** y = 5.3821748∙10^-13^x^5^ – 8.6679154994∙10^-10^x^4^ + 7.0215619042691∙10^-7^x^3^ - 2.0418966137292200∙10^-4^x^2^ + 0.0133777521406273x + 21.7643771919129

R² = 0.82, p-value: 1.8e^-10^

**45%:** y = -2.2904374004∙10^-10^x^4^ + 3.9021701365993∙10^-7^x^3^ - 1.3378135853359110^-4^x^2^ + 7.11410447819105∙10^-3^x + 22.0576792587603

R² = 0.83, p-value: 1.4e^-11^

**55%:** y = -7.89856874∙10^-12^x^5^ + 6.27778052893∙10^-9^x^4^ – 1.40461724204566∙10^-6^x^3^ + 4.84932614366329∙10^-5^x^2^ + 3.03868257719841∙10^-3^x + 22.1601707704622

R² = 0.84, p-value: 2.1e^-11^

**65%:** y = -7.1582855494∙10^-10^x^4^ + 7.3249347776281∙10^-7^x^3^ – 2.01722512451319∙10^-4^x^2^ + 9.69801123460456∙10^-3^x + 22.6333937871236

R² = 0.93, p-value: < 2.2e^-16^

**75%:** y = -4.44808908∙10^-12^x^5^ + 2.69906430718∙10^-9^x^4^ – 5.213565860973∙10^-8^x^3^ – 1.63693355725414∙10^-4^x^2^ + 0.0135403068244386x + 22.8101181285683

R² = 0.91, p-value: < 2.9e^-15^

**85%:** y = -2.98502355∙10^-12^x^5^ + 8.3204818832∙10^-10^x^4^ + 8.1961142721354∙10^-7^x^3^ – 3.47578242561042∙10^-4^x^2^ + 2.98496198269049∙10^-2^x + 22.7277314181438

R² = 0.91, p-value: < 3.2e^-15^

**95%:** y = -5.33250272∙10^-12^x^5^ + 2.77711614452∙10^-9^x^4^ + 3.3896235911157∙10^-7^x^3^ – 3.03409602835814∙10^-4^x^2^ + 2.47593032436271∙10^-2^x + 24.154206900288

R² = 0.97, p-value: < 2.2e^-16^
